# Supplementary material for: Robust switches in thalamic network activity require a timescale separation between sodium and T-type calcium channel activations
Source: PLoS Comput Biol. 2021 May 18;17(5):e1008997. doi: 10.1371/journal.pcbi.1008997 (PMC8162675; doi:10.1371/journal.pcbi.1008997)
Supplement: S1 Supplementary Material — : Quantification of the firing pattern properties. B: Simulation of the reduced models not exhibited in the Results. C: Model description and their parameter values. D: Ionic channel description: steady-state functions and time constants of the gating variables. E: Description of reduced models and their parameter values. (PDF) [file pcbi.1008997.s009.pdf]

Robust switches in thalamic network activity require a timescale separation between sodium and T-type calcium channel activations

Kathleen Jacquerie and Guillaume Drion
Department of Electrical Engineering and Computer Science, University of Liege, Belgium

S1 Supplementary Material

All simulations were performed using the Julia programming language. Analysis were performed either in Matlab and Excel . Code files are freely available at `http://www.montefiore.ulg.ac.be/~guilldrion/Files/Jacquerie2021_codes.zip` and and `https://osf.io/sth4d/`.

A. Quantification of the firing pattern properties
B. Simulations of the reduced models
C. Model description and their parameter values
D. Ionic channel description: steady-state functions and time constants of the gating variables
E. Description of the reduced models and their parameter values

A. Quantification of the firing pattern properties

In Fig 2 (in the main article), for each model, one thousand circuits of two interconnected neurons are generated with random ionic conductances varying from 10, 20 and 30% from their nominal values. Fig S9. 1 shows the spiking frequency in tonic mode (blue) and the intra-burst frequency (orange) among the rhythmic circuits. Models with a fast activation of T-type calcium channels show large variations in the properties of the rhythm. Restoring the slow CaT channel activation reduces the standard deviation (see models 5 vs 5' and models 6 vs 6').

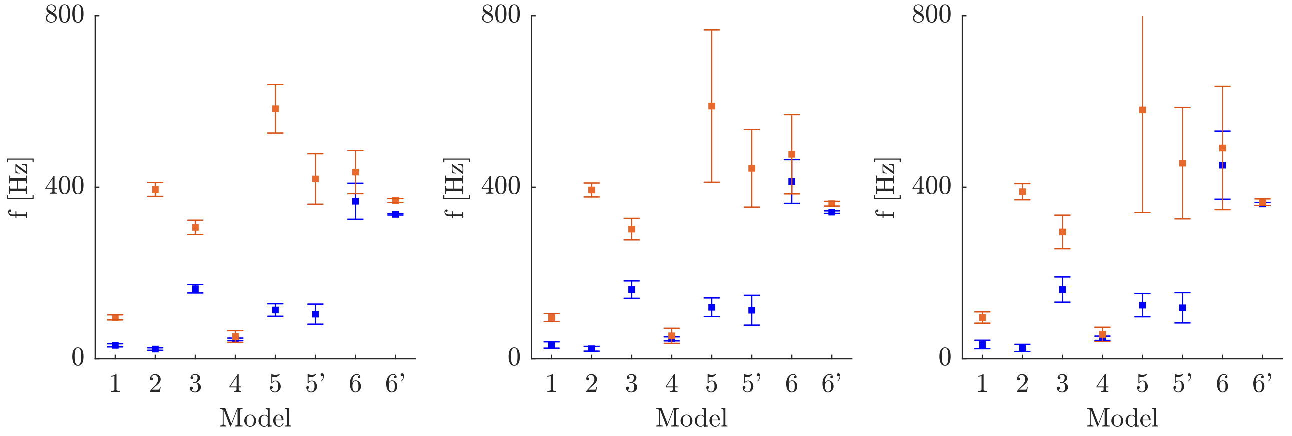

Fig S9. 1: Quantification of the rhythmic networks obtained in Fig 2 (main article) for a variability equals to 10% (left), 20% (center) and 30% (right). The figure plots he mean and the standard deviation of the spiking frequency in tonic mode (blue) and the intraburst frequency (orange) of the inhibitory cell.

B. Simulations of the reduced models

Fig S9. 2 illustrates the simulation of the reduced models not shown in Fig 6 and Fig 7 (in the main article). The top traces shows the switch from tonic to burst when the current is hyperpolarized. The four models have the same behavior (top). However, models embedding a *slow* T-type calcium channels exhibit a *lower branch* in the V-nullcline (models 2, 5' and 6') while model 5 considering an instantaneous activation exhibits N-shape.

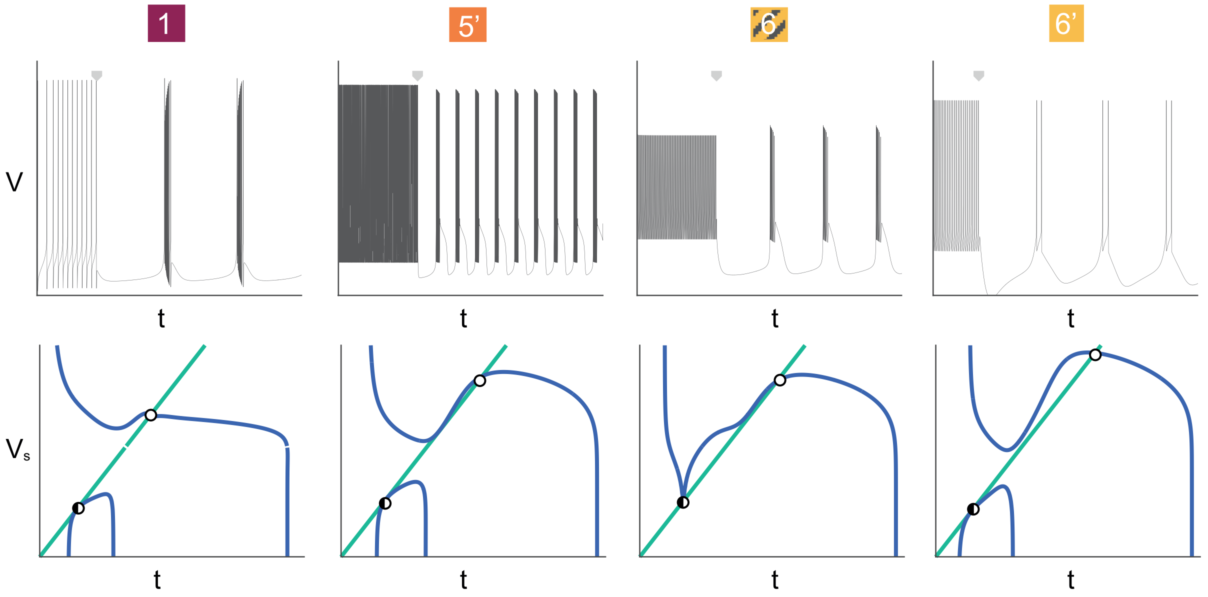

Fig S9. 2: Simulations of the reduced models (top) Voltage recordings of a hyperpolarizing-induced bursting considering its nominal T-type calcium channel activation and (bottom) the associated phase portrait at the saddle node bifurcation (in models 2,5',6 and 6'). The square arrow indicates the hyperpolarizing step current. V-(resp Vs-) nullcline is marked in blue (resp. green). The unstable fixed nodes are drawn in open circles. The half-open circle indicates the meeting between the saddle node and the stable fixed point.

C. Model description and their parameter values

Model 1

Paper: [Drion, Dethier, Franci, and Sepulchre (2018)]

Ionic currents
This model is composed of a six ionic currents:

- a transient sodium current: $I_{Na} = \bar{g}_{Na} m_{Na}^3 h_{Na}(V_m - V_{Na})$,
- a delayed-rectifier potassium current: $I_{K,D} = \bar{g}_{K,D} m_{K,D}^4 (V_m - V_K)$,
- a T-type calcium current: $I_{CaT} = \bar{g}_{CaT} m_{CaT}^3 h_{CaT}(V_m - V_{Ca})$,
- a calcium-activated potassium current: $I_{K,Ca} = \bar{g}_{K,Ca} m_{K,Ca\infty}(Ca)(V_m - V_K)$,
- a hyperpolarization-activation cation current: $I_H = \bar{g}_H m_H (V_m - V_H)$,
- a leak current: $I_{leak} = \bar{g}_{leak}(V_m - V_{leak})$.

Voltage-dependent steady-state functions and associated time constants for activation and inactivation gating variables are given in Table S9. 2. The calcium-dependent activation of the calcium-activated potassium current is modeled as follows: $m_{K,Ca,\infty}([Ca]) = ([Ca]/([Ca]+K_D))^2$. The calcium dynamics

follows the equation $[\dot{Ca}] = -k_1 I_{CaT} - k_2[Ca]$ where k_1 and k_2 are rate parameters. Parameters used in simulations are: $C_m = 1[\mu F/cm^2]$, $V_{Na} = 50$mV, $V_K = -85$mV, $V_{Ca} = 120$mV, $V_{leak} = -55$mV, $V_H = -20$mV, $K_D = 170$. The maximal conductances are expressed in $[mS/cm^2]$ and their nominal values are $g_{Na} = 170$, $\bar{g}_{K,D} = 40$, $\bar{g}_{CaT} = 0.55$, $\bar{g}_{K,Ca} = 4$, $\bar{g}_H = 0.01$ and $g_{leak} = 0.055$

Connectivity
For circuit and network models, the nominal values of maximal synaptic conductances are equal to: $\bar{g}_{\text{AMPA}} = 0.1/n_E$, $\bar{g}_{\text{GABA}_\text{A}} = 0.4/n_I$ and $\bar{g}_{\text{GABA}_\text{B}} = 2/n_I$ where n_E and n_I are respectively the number of excitatory and inhibitory cells.

Applied currents
- At single-cell level, a hyperpolarized-induced bursting is obtained with the applied current equals to 1 then -0.9 $[\mu A/cm^2]$ (referring to Figure 1A).
- Single-cell robustness in C_m; the applied current during the first period is equal to 1 and then it is swept from 0 to -2 with a step of 0.1. The hyperpolarized current leading to the highest robustness in capacitance value variation is -1 (referring to Figure 1C).
- For 2-cell circuit or 200-cell network , the applied current exerted on the inhibitory cells is switching from 1 to -2.6 $[\mu A/cm^2]$ (referring to Figures 2 to 5).

Threshold voltage
In Figure 3B, C and D, the voltage-dependent time constants of sodium channel activation $\tau_{m_{Na}}(V_m)$ and T-type calcium channel activation $\tau_{m_{CaT}}(V_m)$ are evaluated at $V_m = -50mV$. The T-type calcium channel inactivation $\tau_{h_{CaT}}(V_m)$ is evaluated at $V_m = -70mV$.

Currentscapes
Figure 1B shows the switch from tonic to burst in a neuron model whose parameters are: C =1, $\bar{g}_{Na}$ = 200, $\bar{g}_K$ = 20, $\bar{g}_{CaT}$ = 0.75, $\bar{g}_{KCa}$ = 4, $\bar{g}_H$ = 0.1 (top)/$\bar{g}_H$ = 0.0008 (bottom), g_{leak} = 0.055, . The current in the depolarized state is equal to 1 and in the hyperpolarized state -1.85 (top) / -1.07 (bottom). The other parameters remain the same as in the initial description.

Model 2

Paper: [Destexhe, Contreras, Steriade, Sejnowski, and Huguenard (1996)]

Ionic currents
The model is composed of four ionic currents:

- a sodium current: $I_{Na} = \bar{g}_{Na} m_{Na}^3 h_{Na}(V_m - V_{Na})$,
- a potassium current: $I_K = \bar{g}_K m_K^4 (V_m - V_K)$,
- a T-type calcium current: $I_{CaT} = \bar{g}_{CaT} m_{CaT}^2 h_{CaT}(V_m - V_{Ca})$,
- a leakage current: $I_l = \bar{g}_{leak}(V_m - V_{leak})$.

Voltage-dependent steady-state functions and associated time constants for activation and inactivation gating variables are given in Table S9. 3 with $V_2(V) = V - V_{Traub}$. Parameters used in simulations are: $C_m = 1e-3$ $[mF/cm^2]$, $V_{Na} = 50$mV, $V_K = -100$mV, $V_{Ca} = 120$mV, $V_{leak} = -82$mV, $V_{Traub} = -63$mV. Here, we chose to fix the reversal potential of calcium channel rather than evaluate it through calcium concentrations. The maximal conductances are expressed in $[S/cm^2]$ and their nominal values are $\bar{g}_{Na} = 0.4$, $\bar{g}_{K,D} = 0.08$, $\bar{g}_{CaT} = 0.006$, $\bar{g}_{leak} = 5e-5$.

Connectivity
For circuit and network models, the nominal values of maximal synaptic conductances are equal to: $\bar{g}_{\text{AMPA}} = 0.1e^{-3}/n_E$, $\bar{g}_{\text{GABA}_\text{A}} = 0.2e^{-3}/n_I$ and $\bar{g}_{\text{GABA}_\text{B}} = 1e^{-3}/n_I$ where n_E and n_I are respectively

the number of excitatory and inhibitory cells.

Applied currents
- At single-cell level, a hyperpolarized-induced bursting is obtained with the applied current equals to $0.4e^{-3}$ then 0 $[mA/cm^2]$ (referring to Figure 1A).
- Single-cell robustness in C_m; the applied current during the first period is equal to $0.4e^{-3}$ and then it is swept from $0.25e^{-3}$ to -$0.25e^{-3}$ with a step of $0.15e^{-3}$. The hyperpolarized current leading to the highest robustness in capacitance value variation is $0.1e^{-3}$. (referring to Figure 1C).
- For a 2-cell circuit or 200-cell network, the applied current exerted on the inhibitory cells is switching from $0.4e^{-3}$ to -$0.3e^{-3}$ $[mA/cm^2]$ (referring to Figures 2 to 5).

Threshold voltage
In Figure 3B, C and D, the voltage-dependent time constants of sodium channel activation $\tau_{m_{Na}}(V_m)$ and T-type calcium channel activation $\tau_{m_{CaT}}(V_m)$ are evaluated at $V_m = -60mV$. The T-type calcium channel inactivation $\tau_{h_{CaT}}(V_m)$ is evaluated at $V_m = -70mV$.

Model 3

Paper: [Destexhe, Neubig, Ulrich, and Huguenard (1998)]

Ionic currents
The model is composed of four ionic currents:

- a sodium current: $I_{Na} = \bar{g}_{Na} m_{Na}^3 h_{Na}(V_m - V_{Na})$,
- a potassium current: $I_K = \bar{g}_K m_K^4 (V_m - V_K)$,
- a T-type calcium current: $I_{CaT} = \bar{g}_{CaT} m_{CaT}^2 h_{CaT}(V_m - V_{Ca})$,
- a leakage current: $I_l = \bar{g}_{leak}(V_m - V_{leak})$.

Voltage-dependent steady-state functions and associated time constants for activation and inactivation gating variables are given in Table S9.4 with $V_2(V) = V - V_{Traub}$ and a voltage-shift of 3mV for calcium current as explained in [Destexhe et al. (1998)]. Parameters used in simulations are: $C_m = 0.88$ $[\mu F/cm^2]$, $V_{Na} = 50$mV, $V_K = -100$mV, $V_{Ca} = 120$mV, $V_{leak} = -70$mV, $V_{Traub} = -52$mV. Here, we chose to fix the reversal potential of calcium channel instead of computing it through calcium concentration and to use a maximum conductance instead of a maximum permeability. The maximal conductances are expressed in $[mS/cm^2]$ and their nominal values are $\bar{g}_{Na} = 100$, $\bar{g}_{K,D} = 100$, $\bar{g}_{CaT} = 3.3$, $\bar{g}_{leak} = 5e-2$.

Connectivity
For circuit and network models, the nominal values of maximal synaptic conductances are equal to: $\bar{g}_{\mathrm{AMPA}} = 0.1/n_E$, $\bar{g}_{\mathrm{GABA_A}} = 0.2/n_I$ and $\bar{g}_{\mathrm{GABA_B}} = 1/n_I$ where n_E and n_I are respectively the number of excitatory and inhibitory cells.

Applied currents
- At single-cell level, a hyperpolarized-induced bursting is obtained with the applied current equals to 1.5 then -0.7$[\mu A/cm^2]$ (referring to Figure 1A).
- Single-cell robustness in C_m; the applied current during the first period is equal to 1.5 and then it is swept from -0.1 to -1 with a step of 0.1. The hyperpolarized current leading to the highest robustness in capacitance value robustness is -0.6 (referring to Figure 1C).
- For 2-cell circuit or 200-cell network, the applied current exerted on the inhibitory cells is switching from 1.5 to -1.7 $[\mu A/cm^2]$ (referring to Figures 2 to 5).

Threshold voltage
In Figure 3B, C and D, the voltage-dependent time constants of sodium channel activation $\tau_{m_{Na}}(V_m)$ and T-type calcium channel activation $\tau_{m_{CaT}}(V_m)$ are evaluated at $V_m = -40mV$. The T-type calcium channel inactivation $\tau_{h_{CaT}}(V_m)$ is evaluated at $V_m = -60mV$.

Model 4

Papers: [Huguenard and McCormick (1992); McCormick and Huguenard (1992)]

Ionic currents
The model is composed of eleven ionic currents:

- a transient sodium current: $I_{Na} = \bar{g}_{Na} m_{Na}^3 h_{Na}(V_m - V_{Na})$,
- a persistent sodium current: $I_{Nap} = \bar{g}_{Nap} m_{Nap}(V_m - V_{Na})$,
- a T-type calcium current: $I_{CaT} = \bar{g}_{CaT} m_{CaT}^2 h_{CaT}(V_m - V_{Ca})$,
- a calcium-activated potassium current: $I_C = \bar{g}_C m_C(V_m - V_K)$,
- a low threshold calcium current: $I_L = \bar{g}_L m_L^2(V_m - V_{Ca})$,
- several potassium current: $I_{K_{2a}} = \bar{g}_{K_{2a}} m_{K_2} h_{K_{2,a}}(V_m - V_K)$, $I_{K_{2b}} = \bar{g}_{K_{2b}} m_{K_2} h_{K_{2,b}}(V_m - V_K)$, $I_A = \bar{g}_A m_A^4 h_A(V_m - V_K)$, $I_{A_2} = \bar{g}_{A_2} m_{A_2}^4 h_{A_2}(V_m - V_K)$
- a sodium leakage current: $I_{Na,leak} = \bar{g}_{Na,leak}(V_m - V_{Na})$,
- a potassium leakage current: $I_{K,leak} = \bar{g}_{K,leak}(V_m - V_K)$.

Voltage-dependent steady-state functions and associated time constants for activation and inactivation gating variables are given in Table S9. 5. Parameters used in simulations are: $C_m = 0.29$ $[nF]$, $V_{Na} = 50$mV, $V_K = -105$mV, $V_{Ca} = 120$mV. Here, we chose to fix the reversal potential of calcium channel instead of computed it through calcium concentration. The maximal conductances are expressed in $[mS]$ and their nominal values are $\bar{g}_{Na} = 12$, $\bar{g}_{Na_p} = 7e-3$, $\bar{g}_A = 20e-3$, $\bar{g}_{A_2} = 15e-3$, $\bar{g}_{CaT} = 1$, $\bar{g}_{K_{2,a}} = 38e-3$, $\bar{g}_{K_{2,b}} = 26e-3$, $\bar{g}_C = 1$, $\bar{g}_L = 0.8$, $\bar{g}_{Na,leak} = 2.65e-3$ and $\bar{g}_{K,leak} = 7e-3$.

Connectivity
For circuit and network models, the nominal values of maximal synaptic conductances are equal to: $\bar{g}_{\mathrm{AMPA}} = 0.01/n_E$, $\bar{g}_{\mathrm{GABA_A}} = 0.04/n_I$ and $\bar{g}_{\mathrm{GABA_B}} = 0.01/n_I$ where n_E and n_I are respectively the number of excitatory and inhibitory cells.

Applied currents
- At single-cell level, a hyperpolarized-induced bursting is obtained with the applied current equals to 1 then 0.1 $[\mu A/cm^2]$ (referring to Figure 1A).
- Single-cell robustness in C_m; the applied current during the first period is equal to 1 and then it is swept from 0.4 to -0.2 with a step of 0.1. The hyperpolarized current leading to the highest robustness in capacitance value variation is 0.1 (referring to Figure 1C).
- For 2-cell circuit or 200-cell network, the applied current exerted on the inhibitory cells is switching from 1 to 0 $[\mu A/cm^2]$ (referring to Figures 2 to 5).

Threshold voltage
In Figure 3B, C and D, the voltage-dependent time constants of sodium channel activation $\tau_{m_{Na}}(V_m)$ and T-type calcium channel activation $\tau_{m_{CaT}}(V_m)$ are evaluated at $V_m = -50mV$. The T-type calcium channel inactivation $\tau_{h_{CaT}}(V_m)$ is evaluated at $V_m = -60mV$.

Model 5

Paper: [Wang (1994)]

Ionic currents
This model is composed of a six ionic currents:

- a transient sodium current: $I_{Na} = \bar{g}_{Na} m^3_{Na,\infty}(0.85 - m_K)(V_m - V_{Na})$,
- a persistent sodium current: $I_{Nap} = \bar{g}_{Nap} m^3_{Nap,\infty}(V_m - V_{Na})$,
- a potassium current: $I_K = \bar{g}_K m^4_K (V_m - V_K)$,
- a T-type calcium current: $I_{CaT} = \bar{g}_{CaT} m^3_{CaT,\infty} h_{CaT}(V_m - V_{Ca})$,
- a sag current: $I_H = \bar{g}_H m^2_H (V_m - V_H)$,
- a leak current: $I_{leak} = \bar{g}_{leak}(V_m - V_{leak})$.

Voltage-dependent steady-state functions and associated time constants for activation and inactivation gating variables are given in Table S9. 6. Parameters used in simulations are: $C_m = 1[\mu F/cm^2]$, $V_{Na} = 55$mV, $V_K = -80$mV, $V_{Ca} = 120$mV, $V_{leak} = -70$mV, $V_H = -40$mV, $\sigma_K = 10$, $\sigma_{Na} = 6$, $\sigma_{NaP} = -5$, $\theta_h = -79$ and $k_h = 5$. The maximal conductances are expressed in $[mS/cm^2]$ and their nominal values are $\bar{g}_{Na} = 42$, $\bar{g}_{Nap} = 9$, $\bar{g}_K = 30$, $\bar{g}_{CaT} = 1$, $\bar{g}_H = 0.04$ and $\bar{g}_{leak} = 0.12$

Connectivity
For circuit and network models, the nominal values of maximal synaptic conductances are equal to: $\bar{g}_{\text{AMPA}} = 0.1/n_E$, $\bar{g}_{\text{GABA}_\text{A}} = 0.4/n_I$ and $\bar{g}_{\text{GABA}_\text{B}} = 4/n_I$ where n_E and n_I are respectively the number of excitatory and inhibitory cells.

Applied currents
- At single-cell level, a hyperpolarized-induced bursting is obtained with the applied current equals to 3 then -1.3 $[\mu A/cm^2]$ (referring to Figure 1A).
- Single-cell robustness in C_m; the applied current during the first period is equal to 3 and then it is swept from -0.2 to -2 with a step of 0.2. The hyperpolarized current leading to the highest robustness in capacitance value robustness is -1.5 (referring to Figure 1C).
-For 2-cell circuit or 200-cell network, the applied current exerted on the inhibitory cells is switching from 3 to -1.3 $[\mu A/cm^2]$(referring to Figures 2 to 5).

Model 5′

The model designed by Wang in 1994 [Wang (1994)] has set the activation variable of the calcium channel as its steady-state value. It means this activation happens instantaneously without any dynamics *i.e.* without any time constant. We build a new version of Wang model and call it WangCa or model 5′. We restore the slow activation of this channel by integrating the initial expression of the channel as described in the Wang model designed in 1991 with $V_s = 2$. The time constant is equal to:

$$\tau_{m_{CaT}} = 2.5 \frac{1.7 + \exp\left[\frac{-(V+V_s+28.8)}{13.5}\right]}{1 + \exp\left[\frac{-(V+V_s+63)}{7.8}\right]}$$

The expression is slightly scaled compared to the original one in [Wang, Rinzel, and Rogawski (1991)]. The time constant of the calcium channel inactivation is also scaled with a factor 5 ($\tau_{h_{CaT}}[WangCa] = 5\tau_{h_{CaT}}[Wang]$). The other parameters remain the same except in Figure 1C, the hyperpolarizing current leading the highest robustness in capacitance value variation is equal to -1.9 (instead of -1.5).

Threshold voltage
In Figure 3B, C and D, the voltage-dependent time constants of sodium channel activation $\tau_{m_{Na}}(V_m)$ and T-type calcium channel activation $\tau_{m_{CaT}}(V_m)$ are evaluated at $V_m = -40mV$. The T-type calcium channel inactivation $\tau_{h_{CaT}}(V_m)$ is evaluated at $V_m = -60mV$.

Model 6

Paper: [Rush and Rinzel (1994)]

Ionic currents
This model is composed of a five ionic currents:

- a transient sodium current: $I_{Na} = \bar{g}_{Na} m^3_{Na,\infty}(V_m)(0.85 - m_K)(V_m - V_{Na})$,
- a potassium current: $I_K = \bar{g}_K m^4_K (V_m - V_K)$,
- a T-type calcium current: $I_{CaT} = \bar{g}_{CaT} m^3_{CaT,\infty} h_{CaT}(V_m - V_{Ca})$,
- a sodium leak current: $I_{Naleak} = \bar{g}_{Naleak}(V_m - V_{Na})$,
- a sodium leak current: $I_{Kleak} = \bar{g}_{Kleak}(V_m - V_K)$.

Voltage-dependent steady-state functions and associated time constants for activation and inactivation gating variables are given in Table S9. 7. Parameters used in simulations are:$C_m = 1[\mu F/cm^2]$, $V_{Na} = 50$mV, $V_K = -85$mV, $V_{Ca} = 120$mV, $\theta_s = -63$, $k_s = -7.8$, $\theta_h = -72$, $k_h = 1.1$,$\sigma_m = 10.3$, $\sigma_n = 9.3$ and $\phi = 1$. The maximal conductances are expressed in $[mS/cm^2]$ and their nominal values are $\bar{g}_{Na} = 120$, $\bar{g}_K = 10$, $\bar{g}_{CaT} = 0.3$, $\bar{g}_{Naleak} = 0.01429$ and $\bar{g}_{Kleak} = 0.08571$

Connectivity
For circuit and network models, the nominal values of maximal synaptic conductances are equal to: $\bar{g}_{\mathrm{AMPA}} = 0.1/n_E$, $\bar{g}_{\mathrm{GABA_A}} = 0.4/n_I$ and $\bar{g}_{\mathrm{GABA_B}} = 2/n_I$ where n_E and n_I are respectively the number of excitatory and inhibitory cells.

Applied currents
- At single-cell level, a hyperpolarized-induced bursting is obtained with the applied current equals to 15 then -1.2 $[\mu A/cm^2]$ (referring to Figure 1A).
- Single-cell robustness in C_m; the applied current during the first period is equal to 15 and then it is swept from -0.6 to -1.3 with a step of 0.1. The hyperpolarized current leading to the highest robustness in capacitance value variation is -1.2 (referring to Figure 1C).
- For 2-cell circuit or 200-cell network, the applied current exerted on the inhibitory cells is switching from 15 to -1.2 $[\mu A/cm^2]$ (referring to Figures 2 to 5).

Model 6′

We built is a modified version of the neuron model established by Rush and Rinzel 1994 [Rush and Rinzel (1994)]. While the activation of calcium current was considered as an instantaneous event in [Rush and Rinzel (1994)], we restore the slow activation of the T-type calcium channel. We call this new model RushCa or model 6′. To do so, we added a voltage-dependent function for the time constant of the T-type calcium channel activation and remove the simplification that was performed in the original paper. Therefore, the ionic currents are the same as the initial model except for the

T-type calcium current: $I_{CaT} = \bar{g}_{CaT} m_{CaT}^3 h_{CaT}(V_m - V_{Ca})$. The associated time constant is expressed as follows:

$$\tau_{m_{CaT}} = 0.1 \frac{1.7 + \exp\left[\frac{-(V+28.8)}{13.5}\right]}{1 + \exp\left[\frac{-(V+63)}{7.8}\right]}$$

The membrane capacitance is reduced by a factor of 10 such as $C_m = 0.1[\mu F/cm^2]$. In order to respect the physiological timescale of the different ionic currents (such as fast activation and slow inactivation of sodium channels, slow activation of potassium channels, slow activation and ultraslow inactivation of calcium channels), we adapted the time constant of the calcium channel inactivation and the potassium channel activation:$\tau_{h_{CaT}}$[RushCa] $= 1.5\tau_{h_{CaT}}$[Rush] and τ_{m_K}[RushCa]$= 0.175\tau_{m_K}$[Rush]. The synaptic conductances remain the same. The external current is also the same for every simulation. Except the hyperpolarized current leading to the highest robustness in capacitance value variation is -1.1 (instead of -1.2).

Threshold voltage

In Figure 3B, C and D, the voltage-dependent time constants of sodium channel activation $\tau_{m_{Na}}(V_m)$ and T-type calcium channel activation $\tau_{m_{CaT}}(V_m)$ are evaluated at $V_m = -40mV$. The T-type calcium channel inactivation $\tau_{h_{CaT}}(V_m)$ is evaluated at $V_m = -50mV$.

Model summary

Model #<br>Model name	1<br>Drion	2<br>Destexhe	3<br>Destexhe 1998	4<br>Huguenard and McCormick	5<br>Wang	6<br>Rush	$5'$<br>WangCa	$6'$<br>RushCa
Publication year	2018	1996	1998	1992	1994	1994	-	-
Number of ionic currents	6	4	4	11	6	5	6	5
T-type calcium channel activation dynamics	slow	slow	slow	slow	instant.	instant.	slow	slow

Table S9. 1: Key-information related to the conductance-based models

D. Ionic channel description: steady-state functions and time constants of the gating variables

I_i	gating variable	time constant
I_{Na}	$m_{Na,\infty} = \dfrac{1}{1+\exp[(V+35.5)/-5.29]}$	$\tau_{m,Na} = 1.32 - \dfrac{1.26}{1+\exp[(V+120)/-25]}$
	$h_{Na,\infty} = \dfrac{1}{1+\exp[(V+48.9)/5.18]}$	$\tau_{h,Na} = \frac{0.67}{1+\exp[(V+62.9)/-10.0]} {}^{*} \left(1.5 + \frac{1}{1+\exp[(V+34.9)/3.6]}\right)$
$I_{K,D}$	$m_{K,D,\infty} = \dfrac{1}{1+\exp[(V+12.3)/-11.8]}$	$\tau_{m,KD} = 7.2 - \dfrac{6.4}{1+\exp[(V+28.3)/-19.2]}$
I_{CaT}	$m_{CaT,\infty} = \dfrac{1}{1+\exp[(V+67.1)/-7.2]}$	$\tau_{m,CaT} = 21.7 - \dfrac{21.3}{1+\exp[(V+68.1)/-20.5]}$
	$h_{CaT,\infty} = \dfrac{1}{1+\exp[(V+80.1)/5.5]}$	$\tau_{h,CaT} = 410 - \dfrac{179.6}{1+\exp[(V+55)/-16.9]}$
I_H	$m_{H,\infty} = \dfrac{1}{1+\exp[(V+80)/6]}$	$\tau_{m,H} = 272 + \dfrac{1149}{1+\exp[(V+42.2)/-8.73]}$

Table S9. 2: Steady-state functions for channel gating variables and time constants for the different ion channels present in Drion model (Model 1).

I_i	gating variable	time constant
I_{Na}	$\alpha_{m_{Na}} = \dfrac{0.32(13-V_2(V))}{\exp\left[\frac{13-V_2(V)}{4}\right]-1}$	$\tau_{m,Na} = \dfrac{1}{\alpha_{m_{Na}}+\beta_{m_{Na}}}$
	$\beta_{m_{Na}} = \dfrac{0.28(V_2(V)-40)}{\exp\left[\frac{V_2(V)-40}{5}\right]-1}$	
	$\alpha_{h_{Na}} = 0.128\exp\left[\frac{17-V_2(V)}{18}\right]$	$\tau_{h,Na} = \dfrac{1}{\alpha_{h_{Na}}+\beta_{h_{Na}}}$
	$\beta_{h_{Na}} = \dfrac{4}{1+exp\left[\frac{40-V_2(V)}{5}\right]}$	
I_K	$\alpha_{m_K} = \dfrac{0.032(15-V_2(V))}{\exp\left[\frac{15-V_2(V)}{5}\right]-1}$	$\tau_{m,K} = \dfrac{1}{\alpha_{m_K}+\beta_{m_K}}$
	$\beta_{m_K} = 0.5\exp\left[\frac{10-V_2(V)}{40}\right]$	
I_{CaT}	$m_{CaT,\infty} = \dfrac{1}{1+\exp\left[\frac{-(V+50)}{7.4}\right]}$	$\tau_{m_{CaT}} = 1 + \dfrac{0.33}{\exp\left[\frac{-(V+100)}{15}\right]+\exp\left[\frac{V+25}{10}\right]}$
	$h_{CaT,\infty} = \dfrac{1}{1+\exp\left[\frac{V+80}{5}\right]}$	$\tau_{h_{CaT}} = 28.3 + \dfrac{0.33}{\exp\left[\frac{V+48}{4}\right]+\exp\left[\frac{-(V+407)}{50}\right]}$

Table S9. 3: Steady-state functions for channel gating variables and time constants for the different ion channels present in Destexhe,1996 model (Model 2).

I_i	gating variable	time constant
I_{Na}	$\alpha_{m_{Na}} = \dfrac{0.32(13 - V_2(V))}{\exp\left[\frac{13-V_2(V)}{4}\right] - 1}$	$\tau_{m,Na} = \dfrac{1}{\alpha_{m_{Na}} + \beta_{m_{Na}}}$
	$\beta_{m_{Na}} = \dfrac{0.28(V_2(V) - 40)}{\exp\left[\frac{V_2(V)-40}{5}\right] - 1}$	
	$\alpha_{h_{Na}} = 0.128 \exp\left[\frac{17-V_2(V)}{18}\right]$	$\tau_{h,Na} = \dfrac{1}{\alpha_{h_{Na}} + \beta_{h_{Na}}}$
	$\beta_{h_{Na}} = \dfrac{4}{1 + exp\left[\frac{40-V_2(V)}{5}\right]}$	
I_K	$\alpha_{m_K} = \dfrac{0.032(15 - V_2(V))}{\exp\left[\frac{15-V_2(V)}{5}\right] - 1}$	$\tau_{m,K} = \dfrac{1}{\alpha_{m_K} + \beta_{m_K}}$
	$\beta_{m_K} = 0.5 \exp\left[\frac{10-V_2(V)}{40}\right]$	
I_{CaT}	$m_{CaT,\infty} = \dfrac{1}{1 + \exp\left[\frac{-(V+59)}{6.2}\right]}$	$\tau_{m_{CaT}} = 0.204 + \dfrac{0.333}{\exp\left[\frac{V+18.8}{18.2}\right] + \exp\left[\frac{-(V+134)}{16.7}\right]}$
	$h_{CaT,\infty} = \dfrac{1}{1 + \exp\left[\frac{V+83}{4}\right]}$	for $V < -80$: $\tau_{h_{CaT}} = 0.33 \exp\left[\frac{V+469}{66.6}\right]$
		for $V \geq -80$: $\tau_{h_{CaT}} = 9.32 + 0.33 \exp\left[\frac{V+24}{10.5}\right]$

Table S9. 4: Steady-state functions for channel gating variables and time constants for the different ion channels present in Destexhe,1998 model (Model 3).

I_i	gating variable	time constant
I_{Na}	$\alpha_{m_{Na}} = \frac{0.091(V+38)}{1-\exp\left[\frac{-(V+38)}{5}\right]}$	$\tau_{m,Na} = \frac{1}{\alpha_{m_{Na}} + \beta_{m_{Na}}}$
	$\beta_{m_{Na}} = \frac{-0.062(V+38)}{1-\exp\left[\frac{V+38}{5}\right]}$	
	$\alpha_{h_{Na}} = 0.016\exp\left[\frac{-(V+55)}{15}\right]$	$\tau_{h,Na} = \frac{1}{\alpha_{h_{Na}} + \beta_{h_{Na}}}$
	$\beta_{h_{Na}} = \frac{2.07}{\exp\left[\frac{17-V}{21}\right]+1}$	
I_{Nap}	$m_{Nap,\infty} = \frac{1}{1+\exp\left[\frac{-(V+49)}{5}\right]}$	$\alpha_{m_{Nap}} = \frac{0.091(V+38)}{1-\exp\left[\frac{-(V+38)}{5}\right]}$, $\beta_{m_{Nap}} = \frac{-0.062(V+38)}{1-\exp\left[\frac{V+38}{5}\right]}$
		$\tau_{m,Nap} = \frac{1}{\alpha_{m_{Nap}} + \beta_{m_{Nap}}}$
I_L	$\alpha_{m_L} = \frac{1.6}{1+\exp[-0.072(V-5)]}$	$\tau_{m,L} = \frac{1}{\alpha_{m_L} + \beta_{m_L}}$
	$\beta_{m_L} = \frac{0.02(V-1.31)}{\exp\left[\frac{V-1.31}{5.36}\right]-1}$	
I_C	$\alpha_{m_C} = 2.5e5\,[CaL]\exp(V/24)$	$\tau_{m,C} = \frac{1}{\alpha_{m_C} + \beta_{m_C}}$
	$\beta_{m_C} = 0.1\exp[-V/24]$	
I_{CaT}	$m_{CaT,\infty} = \frac{1}{1+\exp\left[\frac{-(V+57)}{6.2}\right]}$	$\tau_{m_{CaT}} = 0.612 + \frac{1}{\exp\left[\frac{-(V+131.6)}{16.7}\right]+\exp\left[\frac{V+16.8}{18.2}\right]}$
	$h_{CaT,\infty} = \frac{1}{1+\exp\left[\frac{V+81}{4.03}\right]}$	for $V < -80$: $\tau_{h_{CaT}} = \exp\left[\frac{V+467}{66.6}\right]$
		for $V \geq -80$: $\tau_{h_{CaT}} = 28 + \exp\left[\frac{-(V+21.88)}{10.2}\right]$
I_A	$m_{A,\infty} = \frac{1}{1+\exp\left[\frac{-(V+60)}{8.5}\right]}$	$\tau_{m_A} = 0.37 + \frac{1}{\exp\left[\frac{V+35.82}{19.697}\right]+\exp\left[\frac{V+79.69}{-12.7}\right]}$
	$h_{A,\infty} = \frac{1}{1+\exp\left[\frac{V+78}{6}\right]}$	for $V < -63$: $\tau_{h_A} = \frac{1}{\exp\left[\frac{V+46.05}{5}\right]+\exp\left[\frac{V+238.4}{-37.45}\right]}$
		for $V \geq -63$: $\tau_{h_A} = 19$
$I_{A,2}$	$m_{A2,\infty} = \frac{1}{1+\exp\left[\frac{-(V+36)}{20}\right]}$	$\tau_{m_{A2}} = 0.37 + \frac{1}{\exp\left[\frac{V+35.82}{19.697}\right]+\exp\left[\frac{V+79.69}{-12.7}\right]}$
	$h_{A2,\infty} = \frac{1}{1+exp\left[\frac{V+78}{6}\right]}$	for $V < -73$: $\tau_{h_{A2}} = \frac{1}{\exp\left[\frac{V+46.05}{5}\right]+\exp\left[\frac{V+238.4}{-37.45}\right]}$
		for $V \geq -73$ $\tau_{h_{A2}} = 60$
I_{K2}	$m_{K2,\infty} = \frac{1}{1+\exp\left[\frac{V+43}{-17}\right]}$	$\tau_{m_{K2}} = 9.9 + \frac{1}{\exp\left[\frac{V-81}{25.6}\right]+\exp\left[\frac{V+132}{-18}\right]}$
	$h_{K2a,\infty} = \frac{1}{1+\exp\left[\frac{V+58}{10.6}\right]}$	$\tau_{h_{K2a}} = 120 + \frac{1}{\exp\left[\frac{V-1.329}{200}\right]+\exp\left[\frac{V+130}{-7.1}\right]}$
I_{K2b}	$h_{K2b,\infty} = \frac{1}{1+\exp\left[\frac{V+58}{10.6}\right]}$	for $V < -70$: $\tau_{h_{K2b}} = 120 + \frac{1}{\exp\left[\frac{V-1.329}{200}\right]+\exp\left[\frac{V+130}{-7.1}\right]}$
		for $V \geq -70$: $\tau_{h_{K2b}} = 8.9$

Table S9. 5: Steady-state functions for channel gating variables and time constants for the different ion channels present in Huguenard and McCormick model (model 4).

I_i	gating variable	time constant
I_{Na}	$\alpha_{m_{Na}} = \dfrac{-0.1(V+29.7-\sigma_{Na})}{\exp\left[-0.1(V+29.7-\sigma_{Na})\right]-1}$	-
	$\beta_{m_{Na}} = 4\exp\left[\frac{-(V+54.7-\sigma_{Na})}{18}\right]$	
I_{Nap}	$\alpha_{m_{Nap}} = \dfrac{-0.1(V+29.7-\sigma_{Nap})}{\exp\left[-0.1(V+29.7-\sigma_{Nap})\right]-1}$	-
	$\beta_{m_{Nap}} = 4\exp\left[\frac{-(V+54.7-\sigma_{Nao})}{18}\right]$	
I_K	$\alpha_{m_K} = \dfrac{-0.01(V+45.7-\sigma_K)}{\exp\left[-0.1(V+45.7-\sigma_K)\right]-1}$	$\tau_{m,K} = \dfrac{7/200}{\alpha_{m_K}+\beta_{m_K}}$
	$\beta_{m_K} = 0.125\exp\left[\frac{-(V+55.7-\sigma_K))}{80}\right]$	
I_{CaT}	$m_{CaT,\infty} = \dfrac{1}{\exp\left[\frac{-(V+65)}{7.8}\right]}$	-
	$h_{CaT,\infty} = \dfrac{1}{\exp\left[\frac{(V-\theta_h)}{k_h}\right]}$	$\tau_{h,CaT} = \frac{1}{2}\left\{\dfrac{\exp\left[\frac{V+162.3}{17.8}\right]}{\exp\left[\frac{(V-\theta_h)}{k_h}\right]}+20\right\}$
I_H	$m_{H,\infty} = \dfrac{1}{1+\exp\left[\frac{V+69}{7.1}\right]}$	$\tau_{m_H} = \dfrac{1000}{\exp\left[\frac{V+66.4}{9.3}\right]+\exp\left[\frac{-(V+81.6)}{13}\right]}$

Table S9. 6: Steady-state functions for channel gating variables and time constants for the different ion channels present in Wang model (Model 5).

I_i	gating variable	time constant
I_{Na}	$\alpha_{m_{Na}} = \dfrac{0.1(V+35-\theta_m)}{1-\exp\left[-0.1(V+35-\sigma_m)\right]}$	-
	$\beta_{m_{Na}} = 4\exp\left[-0.05(V+60-\sigma_m)\right]$	
I_K	$\alpha_{m_K} = \dfrac{0.01(V+50-\sigma_n)}{1-\exp\left[-0.1(V+50-\sigma_n)\right]}$	$\tau_{m,K} = \dfrac{0.05}{\alpha_{m_K}+\beta_{m_K}}$
	$\beta_{m_K} = 0.125\exp\left[-0.0125(V+60-\sigma_n)\right]$	
I_{CaT}	$m_{CaT,\infty} = \dfrac{1}{\exp\left[\frac{V-\theta_s}{ks}\right]}$	-
	$h_{CaT,\infty} = \dfrac{1}{0.5+\sqrt{0.25+\exp\left[\frac{V-\theta_h}{kh}\right]}}$	$\tau_{h,CaT} = 1/\phi\left\{\dfrac{\exp\left[\frac{V+150}{18}\right]}{1.5+\sqrt{0.25+\exp\left[\frac{V-80}{4}\right]}}+30\right\}$

Table S9. 7: Steady-state functions for channel gating variables and time constants for the different ion channels present in Rush model (Model 6).

E. Description of the reduced models and their parameter values

For each reduced model, we have adapted maximal ionic conductances and applied currents in order to find a set of parameters allowing a switch from tonic to burst. The adapted parameter values are often expressed as the original value times a constant that can be find in the table for a given scaled time constant (written $coef(\eta, \bar{g}_i)$). Units are defined in Supplementary Material C. The applied current is expressed in two terms: I_{app} exerted during the entire simulation and I_{step} added to I_{app} during the hyperpolarized state. The multiplicative factor of the CaT time constant is indicated by η.

Reduced model 1

$C_m = 1$, $V_{Na} = 50$, $V_K = -85$, $V_{Ca} = 120$, $V_{leak} = -55$, $V_H = -20$, $K_d = 170$, $\bar{g}_{leak} = 0.055 * coef(\eta, \bar{g}_{leak})$, $\bar{g}_{Na} = 150$, $\bar{g}_{Kd} = 40$, $\bar{g}_{CaT} = 0.55 * coef(\eta, \bar{g}_{CaT})$,$\bar{g}_{KCa} = 0.4$, $\bar{g}_H = 0.01 * coef(\eta, \bar{g}_H)$,$k_1 = 1.e-1$, $k_2 = 0.1e-1$, $I_{app} = 0$, $I_{step} = coef(\eta, I_{step})$. The values of $coef(\eta, param)$ can be found in Table S9.8.

The differential equation $d[Ca]/dt$ describing the dynamics of the calcium driving the calcium-activated potassium current ($I_{KCa} = \bar{g}_{KCa} m_{KCa}([Ca])(V_m - V_K)$) is replaced by a fixed calcium concentration $[Ca]$ equal to 10.

η	I_{step}	$\bar{g}_{leak}$	$\bar{g}_{CaT}$	$\bar{g}_H$
1/500	-3.3	2	1.9	2
1/200	-3.3	2	1.9	2
1/100	-3.3	2	1.9	2
1/50	-3.2	2	1.9	2
1/20	-2.5	2	1.2	2
1/10	-2.2	2	1.2	2
1/5	-3.3	2	1.2	2
1/2	-3.2	2	1.2	2
1	-2.5	2	1.2	2
2	-2.2	2	1.25	2
5	-2.9	2	1.3	2
10	-3	2	1.3	2
20	-3	2	1.3	2
50	-3	2	1.3	2
100	-3	2	1.3	2

Table S9. 8: Parameters for the reduced model 1 as a function of the multiplicative factor (η) of $\tau_{m_{CaT}}$

Reduced model 2

$V_{Na} = 50; V_K = -100; V_{leak} = -82; V_{Ca} = 120; V_{traub} = -63.; \bar{g}_{Na} = 0.4 * coef(\eta, \bar{g}_{Na}); \bar{g}_K = 0.08 * coef(\eta, \bar{g}_K); \bar{g}_{leak} = 5e-5 * coef(\eta, \bar{g}_{leak}); \bar{g}_{CaT} = 0.006 * coef(\eta, \bar{g}_{CaT}); C_m = 1.e-3; I_{app} = coef(\eta, I_{app}); I_{step} = coef(\eta, I_{step})$. The values of $coef(\eta, param)$ can be found in Table S9.9.

Reduced model 5

$V_{Ca} = 120.; V_H = -40.; V_K = -80.; V_{Na} = 55.; V_{leak} = -70.; \sigma_K = 10.; \sigma_{Na} = 6.; \sigma_{NaP} = -5.; \theta_h = -79.; k_h = 5.; \bar{g}_{CaT} = 1.0 * 1.05; \bar{g}_H = 0.04; \bar{g}_{Kd} = 30.0 * 0.9; \bar{g}_{Na} = 42.0 * 1.1; \bar{g}_{NaP} = 9.0; \bar{g}_{leak} = 0.12; I_{app} = 3.; I_{step} = -4.4$, C_m is equal to 1 (resp. 1/3) in Fig 7 left (resp. right).

Reduced model 5'

$C_m = 1.; V_{Ca} = 120.; V_H = -40.; V_K = -80.; V_{Na} = 55.; V_{leak} = -70.; \sigma_K = 10.; \sigma_{Na} = 6.;$ Ĺ$sigma_{NaP} = -5.; \theta_h = -79.; k_h = 5.; \bar{g}_H = 0.04 * coef(\eta, \bar{g}_H); \bar{g}_{Kd} = 30 * coef(\eta, \bar{g}_{Kd}); \bar{g}_{Na} = 42 * coef(\eta, \bar{g}_{Na}); \bar{g}_{NaP} =$

η	I_{app}	I_{step}	$\bar{g}_{CaT}$	$\bar{g}_{leak}$	$\bar{g}_{Na}$	$\bar{g}_K$
1/20	1.2e-3	-1.4e-3	4.7	2	0.19	0.9
1/10	1.2e-3	-1.3e-3	4.7	2	0.2	0.9
1/5	1.1e-3	-0.8e-3	4	2	0.25	0.9
1/2	1e-3	-0.7e-3	4	2	0.25	0.9
1/1.5	1e-3	-0.9e-3	4	2	0.25	0.9
1	1e-3	-1.1e-3	4	2	0.25	0.9
1.5	1e-3	-1.1e-3	4.1	2	0.25	0.9
2	1e-3	-1.3e-3	4.2	2	0.25	0.9
3	1e-3	-1.4e-3	4.2	2	0.25	0.9
5	1e-3	-1.6e-3	4.4	2.1	0.2	0.7
10	1e-3	-2e-3	4.4	2.1	0.2	0.7
20	1e-3	-2.3e-3	4.4	2	0.25	0.9

Table S9. 9: Parameters for the reduced model 2 as a function of the multiplicative factor (η) of $\tau_{m_{CaT}}$

$9.0 * coef(\eta, \bar{g}_{NaP})$; $\bar{g}_{leak} = 0.12 * coef(\eta, \bar{g}_{leak})$; $\bar{g}_{CaT} = 1.0 * coef(\eta, \bar{g}_{CaT})$; $I_{app} = coef(\eta, I_{app})$, $I_{step} = coef(\eta, I_{step})$. The values of $coef(\eta, param)$ can be found in Table S9.10.

η	I_{app}	I_{step}	$\bar{g}_{Kd}$	$\bar{g}_H$	$\bar{g}_{Na}$	$\bar{g}_{NaP}$	$\bar{g}_{leak}$	$\bar{g}_{CaT}$
1/10000	1	-2.45	0.84	1/1.2	5	0.81	0.35	1.2
1/2000	1	-2.45	0.84	1/1.2	5	0.81	0.35	1.2
1/1000	1	-2.45	0.84	1/1.2	5	0.81	0.35	1.2
1/500	1	-2.45	0.84	1/1.2	5	0.81	0.35	1.2
1/200	1	-2.45	0.84	1/1.2	5	0.81	0.35	1.2
1/100	1	-2.45	0.84	1/1.2	5	0.81	0.35	1.2
1/50	1	-2.45	0.84	1/1.2	5	0.81	0.35	1.2
1/20	1	-2.45	0.84	1/1.2	5	0.81	0.35	1.2
1/10	1	-2.45	0.84	1/1.2	5	0.81	0.35	1.2
1/5	1	-2.45	0.84	1/1.2	5	0.81	0.35	1.2
1/2	1	-2.45	0.84	1/1.2	5	0.81	0.35	1.2
1	1	-2.45	0.84	1/1.2	5	0.81	0.35	1.2
2	0	-2.5	0.65	1.9	5	0.8	0.08	2.4
5	0	-2.5	0.65	1.9	5	0.8	0.08	3
10	0	-2.5	0.65	1.9	5	0.8	0.08	3
20	0	-2.5	0.65	1.9	5	0.8	0.08	3
50	0	-2.5	0.65	1.9	5	0.8	0.08	3
100	0	-2.5	0.65	1.9	5	0.8	0.08	3

Table S9. 10: Parameters for the reduced model 5' as a function of the multiplicative factor (η) of $\tau_{m_{CaT}}$

Reduced model 6

$C_m = 1; V_{Na} = 55; V_K = -85; V_{Ca} = 120; \bar{g}_{Na} = 120; \bar{g}_{Kd} = 10; \bar{g}_{Naleak} = 0.01429; \bar{g}_{Kleak} = 0.08571; \bar{g}_{CaT} = 0.3; \theta_s = -63; k_s = -7.8; \theta_h = -72; k_h = 1.1; \sigma_m = 10.3; \sigma_n = 9.3; I_{app} = 15; I_{step} = -16.2$.

Reduced model 6'

$C_m = 0.1; V_{Na} = 55; V_K = -85; V_{Ca} = 120; \bar{g}_{Na} = 120 * coef(\eta, \bar{g}_{Na}); \bar{g}_{Kd} = 10 * coef(\eta, \bar{g}_{Kd}); \bar{g}_{Naleak} = 0.01429 * coef(\eta, \bar{g}_{Naleak}) \bar{g}_{Kleak} = 0.08571 * coef(\eta, \bar{g}_{Kleak}); \bar{g}_{CaT} = 0.3 * coef(\eta, \bar{g}_{CaT}); \theta_s = -63; k_s =$

$-7.8; \theta_h = -72; k_h = 1.1; \sigma_m = 10.3; \sigma_n = 9.3; I_{app} = coef(\eta, I_{app}); I_{step} = coef(\eta, I_{step})$. The values of $coef(\eta, param)$ can be found in Table S9.11.

η	I_{app}	I_{step}	$\bar{g}_{Na}$	$\bar{g}_K$	$\bar{g}_{Naleak}$	$\bar{g}_{Kleak}$	$\bar{g}_{CaT}$
1/1000	15	-16.2	1	1	1	1	1
1/500	15	-16.2	1	1	1	1	1
1/200	15	-16.2	1	1	1	1	1
1/100	15	-16.2	1	1	1	1	1
1/50	15	-16.2	1	1	1	1	1
1/20	15	-16.2	1	1	1	1	1
1/10	15	-16.2	1	1	1	1	1
1/5	15	-16.2	1	1	1	1	1
1/2	15	-16.2	1	1	1	1	1
1	15	-16.2	1.4	1.1	0.9	1	4
2	15	-16.2	1.4	1.1	0.9	1	4
5	15	-16.2	1.6	1.1	0.8	1	2
10	15	-16.2	1.8	0.85	0.65	1	4
20	15	-16.2	1.8	0.7	0.5	1	5
50	15	-16.2	1.8	0.7	0.5	1	5
100	15	-16.2	1.8	0.7	0.5	1	5

Table S9. 11: Parameters for the reduced model 6' as a function of the multiplicative factor (η) of $\tau_{m_{CaT}}$

References

Destexhe, A., Contreras, D., Steriade, M., Sejnowski, T. J., & Huguenard, J. R. (1996). In vivo, in vitro, and computational analysis of dendritic calcium currents in thalamic reticular neurons. Journal of Neuroscience, 16(1), 169–185. doi: 10.1523/jneurosci.16-01-00169.1996

Destexhe, A., Neubig, M., Ulrich, D., & Huguenard, J. (1998). Dendritic low-threshold calcium currents in thalamic relay cells. Journal of Neuroscience, 18(10), 3574–3588. doi: 10.1523/jneurosci.18-10-03574.1998

Drion, G., Dethier, J., Franci, A., & Sepulchre, R. (2018). Switchable slow cellular conductances determine robustness and tunability of network states. PLoS Computational Biology, 14(4), 1–20. Retrieved from `http://dx.doi.org/10.1371/journal.pcbi.1006125` doi: 10.1371/journal.pcbi.1006125

Huguenard, J. R., & McCormick, D. A. (1992, oct). Simulation of the currents involved in rhythmic oscillations in thalamic relay neurons. Journal of neurophysiology, 68(4), 1373–1383. doi: 10.1152/jn.1992.68.4.1373

McCormick, D. A., & Huguenard, J. R. (1992, oct). A model of the electrophysiological properties of thalamocortical relay neurons. Journal of neurophysiology, 68(4), 1384–1400. doi: 10.1152/jn.1992.68.4.1384

Rush, M. E., & Rinzel, J. (1994, aug). Analysis of bursting in a thalamic neuron model. Biological Cybernetics, 71(4), 281–291. Retrieved from `https://doi.org/10.1007/BF00239616` doi: 10.1007/BF00239616

Wang, X. J. (1994). Multiple dynamical modes of thalamic relay neurons: Rhythmic bursting and intermittent phase-locking. Neuroscience, 59(1), 21–31. doi: 10.1016/0306-4522(94)90095-7

Wang, X. J., Rinzel, J., & Rogawski, M. A. (1991). A model of the T-type calcium current and the low-threshold spike in thalamic neurons. Journal of Neurophysiology, 66(3), 839–850. doi: 10.1152/jn.1991.66.3.839
