# Supplementary material for: Robust switches in thalamic network activity require a timescale separation between sodium and T-type calcium channel activations
Source: PLoS Comput Biol. 2021 May 18;17(5):e1008997. doi: 10.1371/journal.pcbi.1008997 (PMC8162675; doi:10.1371/journal.pcbi.1008997)
Supplement: Attachment — Submitted filename: PCOMPBIOL-D-20-01928_reviewer_report_F.pdf [file pcbi.1008997.s010.pdf]

Dear Editor,

I write to report on the manuscript referenced below,

"*Robust switches in thalamic network activity require a timescale separation between sodium and T-type calcium channel activations*" — PCOMPBIOL-D-20-01928

I enjoyed reading this manuscript. I believe the results are technically correct and that they support the claims in the manuscript. The manuscript is well written, well organized, and the quality of the figures is appropriate. I believe the scope of the study is appropriate, and that the results will be of interest for researchers in computational neuroscience.

I did find a number of issues which should be fixed before publication. Please find a complete report in the next pages. Thank you for the opportunity to review for PLOS Computational Biology.

With best regards,

The reviewer

Summary

The manuscript studies the transition between tonic firing and bursting states in conductance based models of neuronal activity. This is motivated by the activities of thalamic neurons which are known to exhibit such transitions across behavioral states. The study addresses the fact that there is a large variability in neurons at the level of their cellular components, such as ionic channel densities, neuromodulation, etc. The study sheds light onto which of these components are important for exhibiting these transitions in a robust way.

Specifically, the manuscript studies six different models that display the transition from tonic spiking to bursting, and performs a sensitivity analysis. This analysis is repeated for different values of the CaT time constant (tau_CaT). The results show that there is a range of tau_CaT for which robustness to perturbations (ie: changes in maximal conductances, due to neuromodulation or other reasons) is larger. The results show that having a separation between the timescales of CaT and Na activations increases the robustness of the single cell models. The manuscript then explores extensions of these results to the case of larger networks.

—-—-—-—-—-—-—-

Thalamic switch

I believe that it is incorrect to call the phenomenon under study a ‘switch’. The switches I know work as follows: I press a button and my lamp stays on, even after I remove my finger. In this case, the switch is a multistable system, which can be on either position depending of whether it is perturbed by my finger. In the case of the thalamic switch, the transition from spiking to bursting takes place when an external current is injected in the cell. But what happens when this current is removed? I am assuming the cells go back to the spiking mode. I believe that in the case of the thalamic switch, what’s happening is that the system undergoes a bifurcation as Iapp is changed, and in the case of the switches in my home, the systems are multistable, which is a very different thing.

I understand that in the context of neuroscience it may be correct to call this phenomenon a ‘switch’, but I believe it is misleading / incorrect and that it would be useful if the authors could speak to some capacity about this.

Author summary

"These brain states translate a collective activity … " (this sentence is not clear)

Line 47: typo ' , , '
Line 116: sentence is not clear — ''It shows that intrinsic . . . "
Line 141: sentence is not clear — 'This only computational change'
Line 144: maybe rephrase — 'To be more quantitative' (maybe, To quantify this, or even removing that could work)
Line 161: maybe replace 'exerted' by 'injected'

Figure 1,

The injected current must be included in the currentscape. There is too much detail in the figure caption. Some detail should be moved to the main text.

Figure 2,

There is too much detail in the figure caption. Some detail should be moved to the main text.

"*The external current, initially depolarized, transiently hyperpolarizes the inhibitory cell and switches the rhythm of the circuit.*"

What do you mean by 'transiently'?

"*By contrast, the right traces show an example of arrhythmic circuits.*"

I have a problem with calling this arrhythmic. It appears to be a periodic solution.

Line 207: typo, "Indeed, <u>their</u> exists different"
Line 213: You are not taking a partial derivative. Please consider using standard notation (like dot, or d/dt)
Line 225: sentence is not clear — "Despite these differences, we were questioning on the choice made"

Figure 3:

There is too much detail in the figure caption. Some detail should be moved to the main text. For example

"There is not differential equation describing its kinetics, consequently, there is no voltage-dependent time constant. The numbers greatly widely vary between each model. It confirms the quantitative differences between them."

Typo "timescale, none circuit switches”

Line 277: typo — "We were repeating the two" 277 (We repeated?)

Figure 4:

This figure needs work.

"It turns on the mean-field rhythm activity of the population depicted by a stronger synaptic activity on the LFP time course and by the transient high power LFP frequency band on the spectrogram.”

What do you mean by transient? The high frequency band appears to stay there for as long as you are injecting the current.

Panels B and D:

The LFP appears to oscillate before the FFT shows any power in the 5Hz band. Why is this? Are the two plots properly aligned?

The white arrows in B (right) are barely visible and it is not clear what they mean.

In the right panels of both B and D there is a diagonal white line which is clearly an artifact of some sort. Please clarify this and fix the figure.

Line 483: Just provide a citation for Julia.

Line 552: typo "Computational experiment on a 2-cell circuit with a varying the 552 T-type calcium activation time constant “ (maybe remove ‘the’?)
