# Supplementary material for: Robust switches in thalamic network activity require a timescale separation between sodium and T-type calcium channel activations
Source: PLoS Comput Biol. 2021 May 18;17(5):e1008997. doi: 10.1371/journal.pcbi.1008997 (PMC8162675; doi:10.1371/journal.pcbi.1008997)
Supplement: Attachment — Submitted filename: Jacquerie_ResponseReviewers.pdf [file pcbi.1008997.s011.pdf]

**Synthesis**

We would like to thank the editor and reviewers for their constructive and insightful comments, which helped us addressing important issues in the revised version of the manuscript. Answers to reviewer comments are given in red. A new section was added in the Results: "Slow T-type calcium shapes a robust phase portrait". The associated methods, supplementary information and codes were updated.

---

**Reviewer #1**: Comments on the manuscript PCOMPBIOL-D-20-01928 by Jacquerie K and Drion G.

The authors study a possible contribution of the activation kinetics of the low threshold calcium current IT to the robustness of subthreshold oscillations of models of thalamic neurons. The study is justified based on the observation that two previously published models of thalamocortical neurons, in which the activation variable of IT is considered instantaneous, are less robust to parameter variations than models that consider relatively slow, more physiologically plausible, IT activation kinetics to switch between tonic and bursting modes. The effect of changing the time scale of IT activation on the robustness of firing mode switching is systematically explored at the cellular and network levels. Robustness of a given model is, in turn, evaluated for its ability to undergo oscillations after variation of parameters such as global conductance scaling (capacitance variation), several degrees of intrinsic and synaptic conductance variability and homogeneity or heterogeneity of connectivity of an artificial network.

The results show that cell and network oscillatory robustness requires a T type calcium current that activates with kinetics between those of its very slow inactivation and the fast activation of the sodium current. Base on this finding, the authors speculate with a more general principle in which the robustness of transitions between different network rhythms (and consequently different functional states) requires both fast and slow positive feedback mechanisms with timescale differences of about one order of magnitude.

The study is well organized and is clearly presented. I personally appreciated the way the sequential increase in complexity of the computational experiments and results is presented; this facilitates comprehension and readability of the manuscript. In general, I consider this to be a good piece of theoretical work that supports the notion that the activation of IT should be modeled preserving the time dependence that has been determined experimentally. Results presented in figure 3C in which the maximum percentage of rhythmic networks coincide almost perfectly with the "natural" (multiplicative factor of 1) kinetics of τmCAT are striking. As pointed out by the authors, reduction of complex relatively fast processes to instantaneous, while maintaining certain phenomenological accuracy, is computationally convenient and also facilitates the mathematical analysis of neuronal models. However, the results presented by Jacquerie and Drion indicate that important properties such as the robustness of oscillatory mode switching could be compromised by this simplification. It is also possible that the dynamical properties of cells and networks could differ if these reductions are made. In this regard, although is out of the scope of the study, it would be interesting to perform an analysis, similar to that performed in the cited work by Rush and Rinzel (model 6 in the present work), to see if the dynamical (bifurcation) structure still holds after including a non-instantaneous activation of IT (e.g. model 6' in the present work).

Based on this suggestion and reviewer #2's comments, we have added a section dedicated to dynamical system analysis. We hope that this new section helps in outlining the role of a timescale separation between fast and slow ionic currents.

I do not have any substantial comment on the quality, originality and importance of the study. I only suggest that a comment should be included in the discussion about the physiological relevance of the findings. It is known that T type channels are subject to modulation by several signaling cascades and are also targets of several experimental and clinical drugs currently in use. It opens the possibility that modulations that change activation kinetics of T type channels have important consequences on physiological and pathological states (e.g. sleep and epilepsy respectively) that involve oscillations generated, sustained or propagated by the thalamic circuit.

Thank you for your remark. Alteration of the T-type calcium channel kinetics due to drugs or experimental protocols is a nice use of this work to study pathological or physiological state perturbations. As highlighted in our computational experiments, modifying the activation kinetics of this channel can lead undesired behavior, as also seen in epilepsy or drug affecting sleep rhythms.  A comment was added in the revised manuscript based on these citations.

**Reviewer #2:** In this study, the authors examined 6 different computational models of thalamocortical neurons, in order to understand common property which is required for switching between post-inhibitory bursting and tonic states. The time scale separation in sodium and calcium currents was identified as an important property across models. They also identified that robustness of this switching, measured by varying parameters, is higher when kinetics were included in the computational model. While the finding on the common property across models involving time scale separation add incremental value to understanding computational models, the finding about robustness does not seem to have clear implications. Below are main issues which could be addressed, to justify the need for publication in this journal.

1. One of the main results of this study is the demonstration of time scale separation in the calcium and Na channel as an important mechanism across models for switching between bursting and tonic states. Previous models have used this mechanism for the switching, and this study help extend the importance of this property across models. In the same motivation, it would be helpful to obtain a minimal model that include all the required currents for switching.

Further, it would be helpful to describe and classify common biologically relevant processes, eg. progressive buildup of L current is common across all models.

This is a very good comment. Regarding the currents required for switching, models for Destexhe et al. are already quite minimal, as they only incorporate a transient sodium current, a delayed-rectifier potassium current, a T-type calcium current and a leak current. Regarding the common biologically relevant processed, we did not add a classification as the models studied where quite different in their composition. We are however open to any specific suggestion on this part.

2. It would be helpful to examine the time scale separation from dynamical system point.

Specifically, authors could add simple phase space plots of the two currents that shows similar qualitative behavior across different models.

This reviewer's suggestion led to the addition of a dynamical system study (see response to Reviewer #1). We have performed the reduction of the conductance-based models 1,2,5,5',6 and 6' by developing a protocol that computes the contribution of each variable in the fast, slow and ultraslow timescales (following Drion et al., eNeuro, doi:10.1523/ENEURO.0031-14.2015). This permitted to analyze the impact of changing the kinetics of activation of the T-type calcium channel on the phase portrait. The results are highlighted in Fig. 6 and Fig. 7, as well as in videos showing the time-course of the membrane voltage and simultaneously the phase portrait evolution during a switch from tonic to (Supplementary videos S1 to S8). Reduction of models 2, 5, 5', 6 and 6' are available in Supplementary Material S9.

3. With respect to results on robustness, authors first show an increase in robustness when kinetics of is implemented compared to instantaneous assumptions for Ca currents. This finding is not surprising. Instantaneous activation is often chosen to have better understanding of other variables or to improve speed under fixed parameter regime. It is not clear what the authors like to achieve in demonstrating improved robustness with adding dynamical variable to instantaneous assumptions, since the outcome is expected.

The reviewer has highlighted an interesting fact: considering an activation as instantaneous is useful when considering a variable fast enough compared to the others. Two reasons are mentioned, (i) it provides a way to study the instantaneous variable in perspective to the others (ii) it is a common strategy to reduce the number of differential equations and therefore reduce the computational time-cost.

Considering the activation of T-type calcium channels as an instantaneous phenomenon means that the opening of this channel operates as a fast positive feedback. Therefore, this positive feedback sums up with the fast positive feedback provided by the activation of sodium channels. However, this simplification, often encountered in literature, neglects the biological distinction between the kinetics of the activation of sodium channels and the activation of T-type calcium channels. In models 5' and 6', the simple addition of a slow differential equation for the activation of this calcium channel enhances the robustness compared with models 5 and 6. On the other hand, adding a differential equation for sodium channel activation does not improve bursting robustness.

The second reason why we added a slow activation in models 5 and 6 is because these models are already capable of generating a switch to bursting even in the absence of a slow activation, whereas models 1 to 5 do not. We therefore felt that it was important to study the difference in robustness between the two mechanisms.

4. There are some differences in robustness across models (that include kinetics) but the study does not provide no additional understanding of reason for this differences. To name a few, does it arise from higher coupling between slow currents?

The main difference in robustness that we observed is between model 4 and models 1,2 and 3. We hypothesized that this comes from the higher numbers of intrinsic parameters that are perturbed. Indeed, model 4 has more than 10 parameters compared to models 1,2 and 3 that have around 5 tuned parameters. Aside from this difference in the number of conductances, we

indeed did not investigate the reasons of the differences in robustness between models, as we wanted to focus on the effect of changing T-type calcium channel activation in each model. This is however a very interesting, yet non-trivial question that would deserve scrutiny in future work.

do H-current play additional role in improved robustness?

We did not specifically test if the H-current plays an additional role to improve robustness in these models, but the switch to bursting was robust to rather large variations in H-current intrinsic conductance in models 1 and 4, and the H-current is absent in models 2 and 3. From a dynamical viewpoint however, T-type calcium channel activation and H-current activation playing a major role in the slow and ultraslow timescales, respectively, there could be a sweep spot in their interactions for robust bursting.

is the phase space larger for each state or a difference in way the switching happens?

Both phenomena are indeed happening. In models 1 to 4 and at nominal calcium channel activation, the silent phase of the burst sits on a lower branch of the V-nullcline, the spiking phase sits on the N-shaped upper branch and the basin of attraction between the two states is separated by the stable manifold of a saddle point on the lower branch. As calcium channel activation becomes faster, the phase space for the silent phase shrinks as the lower branch reduces in size, and the lower branch eventually disappears, disrupting the ability to create robust busting. In models 5 and 6, bursting in the absence of slow calcium channel activation is generated by another mechanism that does not rely on a lower branch of the V-nullcline but rather on a region of the phase plane where the slow variable becomes faster than the fast variable. Here, the increase in robustness due to the addition of a slow activation variable relies on a switch from one mechanism to the other. These properties are discussed in the new results section.

5. Do other property of the bursting and firing rate (like number of burst vary within the regions of switching)? How much is the variation in these properties?

This is indeed a good question. We have added the variations in tonic spiking frequency and intra-burst frequency in all models in the main text as well as in Supplementary material (Fig S9.1). It shows that properties vary much less in models incorporating slow T-type calcium channel activations, although these models are capable of switching for higher variability in their intrinsic properties.

6. Robustness is helpful in maintain similar qualitative behavior under small changes in neuromodulation or synaptic plasticity. But, it also critical to have neuromodulation change the switching states, such as between awake/sleep transition. It would be useful to identify boundaries of small and large variations.

Robustness of behavior and neuromodulation indeed needs to coexist to create flexible and robust neuronal signaling. Here, we focused on the robustness of the functional switch to fast and localized neuromodulation and synaptic plasticity, making neuromodulation of each state possible without disruption the ability to switch. Neuromodulation indeed also happens at larger amplitude, spatial and time-scales, such as observed in the sleep-wake transition. We did not study such neuromodulation, but our results suggest that it would be compatible with robustness

at the local, fast level by tuning the mean properties of neurons within a network, which is coherent with neuromodulation acting on a larger spatial scale.

7. To study robustness, why was Cm chosen as the parameter?, why not other parameters ?

At the single-cell level, we choose Cm as the parameter for two main reasons. From a physiological viewpoint, changing Cm can either model a change in cell size or shape, or a uniform scaling of all the maximal conductances. Such scaling has been observed experimentally (Schultz et al., PNAS, 104, 13187-13191., 2007) and has been shown to be related to homeostatic mechanisms (O'Leary et al., Neuron, 82(4), 809-821., 2014).

In Fig 2 to 5 (circuit and population levels), each maximal conductance is randomly picked with respect to a uniform distribution in a fixed interval around its nominal value. The interval width defines the variability level, as a percentage around this nominal value. For instance, for an intrinsic variability of 10%, each ionic conductance is selected in the range: $[g_i-0.1g_i; g_i+0.1g_i]$.

8. I or RE cells have many intrinsic properties as well, it is not clear if these were examined.

We used models of both reticular and relay cells to check if the results were robust to differences in intrinsic properties, in particular the timescale of T-type calcium channel inactivation. Results were qualitatively equivalent in both neuron types, but we did not examine the quantitative effect of these differences. The only main difference that we could highlight is that a slower inactivation for T-type calcium channels broadens the possible range for T-type calcium channel activation timescale.

**Reviewer #3**

Dear Editor,
I write to report on the manuscript referenced below,

"Robust switches in thalamic network activity require a timescale separation between sodium and T-type calcium channel activations" — PCOMPBIOLD- 20-01928

I enjoyed reading this manuscript. I believe the results are technically correct and that they support the claims in the manuscript. The manuscript is well written, well organized, and the quality of the figures is appropriate. I believe the scope of the study is appropriate, and that the results will be of interest for researchers in computational neuroscience.

I did find a number of issues which should be fixed before publication. Please find a complete report in the next pages. Thank you for the opportunity to review for PLOS Computational Biology.

With best regards,
The reviewer

**Summary**

The manuscript studies the transition between tonic firing and bursting states in conductance based models of neuronal activity. This is motivated by the activities of thalamic neurons which are known to exhibit such transitions across behavioral states. The study addresses the fact that there is a large variability in neurons at the level of their cellular components, such as ionic channel densities, neuromodulation, etc. The study sheds light onto which of these components are important for exhibiting these transitions in a robust way.

Specifically, the manuscript studies six different models that display the transition from tonic spiking to bursting, and performs a sensitivity analysis. This analysis is repeated for different values of the CaT time constant (tau_CaT). The results show that there is a range of tau_CaT for which robustness to perturbations (ie: changes in maximal conductances, due to neuromodulation or other reasons) is larger. The results show that having a separation between the timescales of CaT and Na activations increases the robustness of the single cell models. The manuscript then explores extensions of these results to the case of larger networks.

------

**Thalamic switch**

I believe that it is incorrect to call the phenomenon under study a 'switch'. The switches I know work as follows: I press a button and my lamp stays on, even after I remove my finger. In this case, the switch is a multistable system, which can be on either position depending of whether it is perturbed by my finger. In the case of the thalamic switch, the transition from spiking to bursting takes place when an external current is injected in the cell. But what happens when this current is removed? I am assuming the cells go back to the spiking mode. I believe that in the case of the thalamic switch, what's happening is that the system undergoes a bifurcation as Iapp is changed, and in the case of the switches in my home, the systems are multistable, which is a very different thing.

I understand that in the context of neuroscience it may be correct to call this phenomenon a 'switch', but I believe it is misleading / incorrect and that it would be useful if the authors could speak to some capacity about this.

The reviewer makes a very interesting comment about the definition of a switch in different fields and how discrepancies in the definitions might lead to confusion. In many engineering fields, a switch is indeed related to multistability, creating the ability to create long lasting changes in behavior with transient inputs. In biology in general, a switch is often defined as an ON-OFF change in behavior in response to some input, with a looser connection to the underlying mechanism. Such switches can be of different kinds depending on the underlying mechanism, which includes e.g. non-hysteretic switches (zero-order ultrasensitivity), hysteretic switches (reversible bistability, i.e. returning to the initial state as the input is released), or irreversible switches (irreversible bistability). In the case of a thalamocortical switch, a switch often loosely describes the ON-OFF change in information processing at the level of the network, which can happen on many timescales and through different mechanisms, including transient inputs currents on short timescales (switches in brain state), or neuromodulatory inputs on longer timescales (sleep-wake cycle). We however agree with the reviewer that it would be a stretch to call any ON-OFF phenomena occurring at a bifurcation point a switch, and the open question on what makes a switch is of great interest.

In our computational experiments (models 1-4, 5' and 6'), $I_{app}$ creates a switch by indirectly affecting the unfolding parameters of a (winged-cusp) singularity, making the system "switch" between two unfolding regions where the dynamical and input-output properties are qualitatively different. In order words, it strongly affects the core geometry of the system. This change creates a robust ON-OFF change in information processing at the cellular and network levels. In models 5 and 6, the system indeed simply undergoes a bifurcation as $I_{app}$ is changed, without affecting the geometry of the system itself, which makes the transition much less robust. Having a more precise definition of what makes a switch in neuronal systems could indeed be a good way to isolate robust mechanisms from un-robust ones.

**Author summary**

"These brain states translate a collective activity . . . " (this sentence is not clear)
We modified this sentence as follows:

"These brain states translate activity patterns of neurons interconnected via synaptic connections."

Line 47: typo ' , , '
We removed the extra-comma

Line 116: sentence is not clear — ''It shows that intrinsic . . . "
We modified this sentence as follows:

"It shows that different combinations of ionic currents can lead to same firing pattern."

Line 141: sentence is not clear — 'This only computational change'
We modified this sentence as follows:

"This only modification is sufficient to recover the desired firing activity, ie. the switch from tonic to burst even in the case when the capacitance membrane is divided by 10"

Line 144: maybe rephrase — 'To be more quantitative' (maybe, To quantify this, or even removing that could work)
We removed the part 'to be more quantitative'.

Line 161: maybe replace 'exerted' by 'injected'
Injected has replaced exerted.

**Figure 1,**
The injected current must be included in the currentscape. There is too much detail in the figure caption. Some detail should be moved to the main text.

Figure captions have been shortened directly in the manuscript. The injected current has been highlighted in the Figure 1.

**Figure 2,**

There is too much detail in the figure caption. Some detail should be moved to the main text.

*"The external current, initially depolarized, transiently hyperpolarizes the inhibitory cell and switches the rhythm of the circuit."*
What do you mean by 'transiently'?
It is true that the word "transiently" brings confusion in the simulation protocol performed. The applied current follows a hyperpolarizing step. "Transiently" is removed and the sentence becomes: 'The external current initially depolarizes the inhibitory cell then it is decreased leading to hyperpolarization of the inhibitory cell. This current step switches the rhythm of the circuit. "

*"By contrast, the right traces show an example of arrhythmic circuits."*
I have a problem with calling this arrhythmic. It appears to be a periodic solution.
It is a good remark. The term arrhythmic seems, indeed, not appropriate to support the message. In Fig 2.A (center), the cellular trace illustrates the typical firing activity present in neurons of the thalamic circuitry, ie the target activity. The term of the Fig 2.A (right) was adapted into asynchronous rhythm.

Line 207: typo, "Indeed, their exists different"
The typo was corrected in the text.

Line 213: You are not taking a partial derivative. Please consider using standard notation (like dot, or d/dt)
The equation was corrected in the text.

Line 225: sentence is not clear — "Despite these differences, we were questioning on the choice made"
We modified this sentence as follows:

"Here, we are investigating the choice made on …"

Figure 3:
There is too much detail in the figure caption. Some detail should be moved to the main text. For example

"There is not differential equation describing its kinetics, consequently, there is no voltage-dependent time constant. The numbers greatly widely vary between each model. It confirms the quantitative differences between them."

Typo "timescale, none circuit switches"
"no circuit switches"

Line 277: typo — "We were repeating the two" 277 (We repeated?)
"we repeated"

Figure 4:
This figure needs work.
Figure 4 has been adapted based on your remarks. We have highlighted the fact the LFP time course was a zoom section on the spectrogram. The voltage traces have been added in each situation to enhance the message of the figure.

"It turns on the mean-field rhythm activity of the population depicted by a stronger synaptic activity on the LFP time course and by the transient high power LFP frequency band on the spectrogram."

What do you mean by transient? The high frequency band appears to stay there for as long as you are injecting the current.
Thank you for the remark. Indeed, "transient" was not the appropriate word to describe figure. We removed the term. As soon as the current is hyperpolarizing, the mean-field activity undergoes a change in rhythmic activity. Removing the term transient helps to avoid misunderstood with the possible transient effect that can arise during a short period directly after the current step.

Panels B and D:

The LFP appears to oscillate before the FFT shows any power in the 5Hz band. Why is this? Are the two plots properly aligned? The white arrows in B (right) are barely visible and it is not clear what they mean.
This LFP signal only shows strong oscillation when the current is hyperpolarizing. The time-evolution of the LFP was zoomed and not aligned vertically with the spectrogram of the LFP. We followed your recommendations and improved Fig.4.

In the right panels of both B and D there is a diagonal white line which is clearly an artifact of some sort. Please clarify this and fix the figure.
It was an artefact from the spectrogram saved in eps. It is fixed in the revised manuscript.

Line 483: Just provide a citation for Julia.
A reference has been added:
Bezanson J, Edelman A, Karpinski S, Shah VB. Julia: A fresh approach to numerical computing. SIAM Review. 2017;59(1):65{98. doi:10.1137/141000671.

Line 552: typo "Computational experiment on a 2-cell circuit with a varying the 552 Ttype calcium activation time constant " (maybe remove 'the'? )
Corresponding corrections have been made in the manuscript.
